# Comparative Study of the Catalytic Activities of Three Distinct Carbonaceous Materials through Photocatalytic Oxidation, CO Conversion, Dye Degradation, and Electrochemical Measurements

**DOI:** 10.1038/srep35500

**Published:** 2016-10-20

**Authors:** Hangil Lee, Yeonwoo Kim, Min Ji Kim, Ki-jeong Kim, Byung-Kwon Kim

**Affiliations:** 1 Department of Chemistry, Sookmyung Women’s University, Seoul 140-742, Republic of Korea; 2 Molecular-Level Interfaces Research Center, Department of Chemistry, KAIST, Daejeon 305-701, Republic of Korea; 3 Beamline Research Division, Pohang Accelerator Laboratory (PAL), Pohang 790-784, Republic of Korea

## Abstract

In order to compare the catalytic activities of reduced graphene oxide (rGO), graphene oxide (GO), and graphene, we conducted oxidation of 2-aminothiophenol (2-ATP) and reduction of nitrobenzene (NB) in their presence by using high-resolution photoemission spectroscopy (HRPES). In addition, we determined conversion rates of CO to CO_2_ in the presence of these catalysts by performing a residual gas analyzer (RGA) under a UHV condition, Orange II and methylene blue degradations UV-vis spectrophotometry, and electrochemistry (EC) measurements in an aqueous solution, as well as by obtaining cyclic voltammograms and determining the change of the condition of electrodes before and after the oxidation of 2-ATP. We found that we can successively fabricate GO (oxidation) and graphene (reduction) from rGO by controlling the oxidation or reduction procedure time and then clearly comparing the critical properties among them as we perform various oxidation and reduction activities.

Carbon-based materials such as reduced graphene oxide (rGO), graphene oxide (GO), and graphene are the most extensively utilized supports for metal-based catalysts and are used on their own as oxidative or reductive catalysts because they provide high surface areas, are resistant to acidic and basic media, and their surfaces can be readily engineered by introducing functional groups^1^,^2^,^3^,^4^,^5^,^6^,^7^,^8^. GO typically contains a variety of oxygen functional groups, which suggests that the electronic structure of graphene could be modified under selective conditions for its use as an oxidative catalyst. Its high surface area and rich surface chemistry means that GO could be useful in heterogeneous catalytic systems^9^,^10^,^11^,^12^,^13^. It has recently been demonstrated that graphene can be used as an effective catalyst in reduction reactions^14^,^15^,^16^. In special cases, it can be useful for electrochemical energy storage and toxic gas conversion technologies as well as fuel cells^17^,^18^. rGO can be also used as an efficient metal-free catalyst for catalytic reactions. rGO has intermediate properties between those of GO and graphene, so it is expected that rGO can lead to a more significant improvement in its catalytic performance as we can easily control the oxidation or reduction processes when we easily fabricate GO and graphene^19^,^20^,^21^,^22^,^23^,^24^.

With this aim in mind, by commencing with an rGO sample, we successfully fabricated GO through an oxidation process and graphene through a reduction process (see the data and results section). We then assessed the capacities of GO, rGO, and graphene to oxidize 2-aminothiophenol (2-ATP) and to reduce nitrobenzene (NB) under UHV conditions with 365 nm UV light illumination to enhance the photocatalytic reaction. Moreover, we also compared their catalytic activities in the conversion of CO to CO_2_ by using residual gas analyzer (RGA) in an ultra-high vacuum (UHV) condition, Orange II and methylene blue degradations by using UV-vis spectrophotometry, and in electrochemistry (EC) under an aqueous solution.

Herein, we precisely compared the morphologies and electronic properties of the rGO, GO, and graphene samples, and assessed their catalytic capacities (oxidation and reduction) by using scanning electron microscopy (SEM), tunneling electron microscopy (TEM), Raman spectroscopy, HRPES, RGA, UV-vis spectrophotometry, and EC measurements.

## Results and Discussion

**Figure 1:**
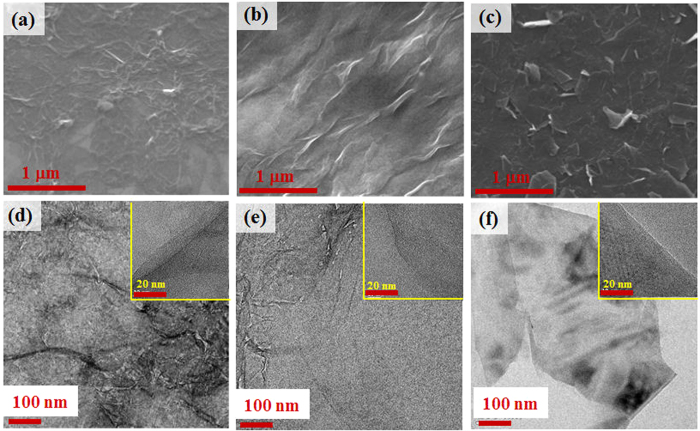
SEM and TEM images of (**a**,**d**) rGO, (**b**,**e**) GO, and (**c**,**f**) graphene, respectively at 300 K.

SEM and TEM were used to characterize the morphologies of the three distinct carbon-based materials (rGO, GO, and graphene). The rGO sample in Fig. 1(a) consists of bundles, as found in previous results^23^. After checking the morphology of rGO, we conducted oxidation and reduction procedures as we controlled the reaction times by 30 minutes to obtain GO particles (using oxidation) and graphene particles (using reduction). The GO-like sample in Fig. 1(b) has a layer-like structure with surface wrinkles due to scrolling and crumpling. This GO-like sample randomly aggregated to form a disordered solid. In addition, as we increased the reduction time, we were able to obtain the graphene-like sheets as shown in Fig. 1(c), which are similar in appearance to those found in previous studies^25^. We also obtained TEM images with the aim of determining the morphological variations between the fabricated rGO, GO, and graphene samples. As shown in Fig. 1(d–f), there are clear differences between the morphological structures of the three distinct carbon-based materials.

**Figure 2:**
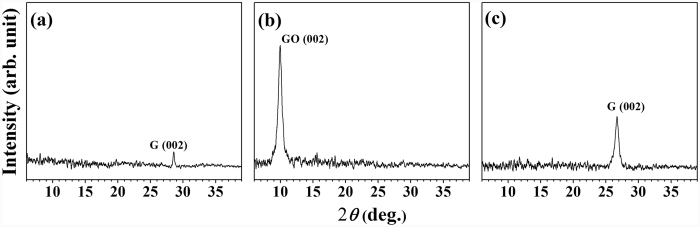
XRD patterns of the fabricated carbon based materials. (**a**) rGO, (**b**) GO, and (**c**) graphene.

**Figure 3:**
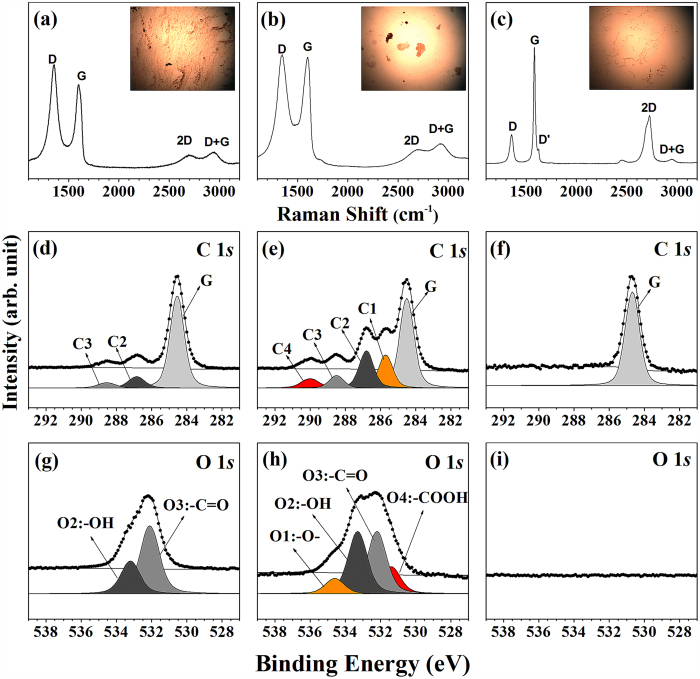
(Top panel) Raman spectra acquired at 300 K of the (**a**) rGO, (**b**) GO, and (**c**) graphene samples spin-coated onto silicon substrates and their corresponding optical images. (Bottom panel) HRPES measurements of the C 1*s* and O 1*s* core level spectra of the (**d,g**) rGO, (**e,h**) GO, and (**f,i**) graphene samples prepared on silicon substrates at 300 K.

Additionally, in order to confirm that the GO and graphene is fabricated from rGO, we also acquired X-Ray diffraction (XRD). As shown in Fig. 2, the XRD patterns of three distinct carbon-based materials (a: rGO, b: GO, and c: graphene) were collected over the range 20°–80° to check the crystalline structures. As shown in Fig. 2, we can clearly compare among them that diffraction peaks at ~10° (GO) and ~26° were observed, which were assigned to the typical (002) peaks of the GO and graphene structure, respectively. All peaks were positioned at 10.0°, 26.7°, 28.5° on GO, Graphene, and rGO, respectively. GO peak was positioned at almost similar position with traditional GO, which represented ~0.88 nm of interlayer distance and somewhat larger due to the synthetic modification of Hummers’ method. Actually, (002) graphene peak is observed at ~23°, however it is very broad due to the small and thin structural size and less crystalline with [002] direction. Therefore, they are not shown in our spectra. And we can observe those structural features by Raman and XPS data in Fig. 3. The rGO and graphene structure obtained at ~27° represent the graphitic structure, which may be indicated from the multilayer stacking of graphene layer during reduction procedure.

Figure 3(a–c) show the Raman spectra of the rGO, GO, and graphene-like samples prepared on silicon substrates. Two notable features of these spectra are apparent in Fig. 3(a,b): the I_D_/I_G_ values of rGO and GO are 1.761 and 0.912 respectively, and the D and G bands at 1347.6 and 1592.1 cm^−1^ are typical of GO and rGO related bands^19^,^26^. The Raman spectrum of the graphene-like sheets is shown in Fig. 3(c). In the Raman spectrum of graphene, the G-peak (1580 cm^−1^) and D-peak (1320 cm^−1^) are due to the *E*_2g_ phonon at the Brillouin zone center and the breathing mode of *sp*^2^ carbon atoms respectively^27^,^28^. Next, we conducted HRPES experiments to compare the electronic structures of the three samples and to determine their carbon contents. The C 1*s* core-level spectra of the rGO (Fig. 3(d)) and GO (Fig. 3(e)) samples contain three and five distinct de-convoluted peaks respectively. The peak center of the binding energy of 284.5 eV (G) was assigned to a C–C bond with a *sp*^2^ character. The other peaks were assigned to epoxy groups (C–O–C, 285.6 eV: C1), hydroxyl groups (–C–OH, 286.7 eV: C2), carbonyl groups (–C=O, 288.5 eV: C3), and carboxyl groups (–COOH, 290.1 eV: C4)^20^. In Fig. 3(f), only a single peak due to the C 1*s* core-level spectrum is evident at 284.7 eV, which corresponds to the binding energy of surface *sp*^2^ hybridized states^29^. This result confirms that our fabricated graphene-like sheets are indeed graphene.

We also acquired O 1*s* core-level spectra of rGO, GO, and graphene-like samples as shown in Fig. 3(g,i). Figure 3(h) shows the O 1*s* core-level spectrum of the GO. The core-level spectra indicated the presence of four functional groups on the GO film: epoxy, hydroxyl, carbonyl, and carboxyl groups. Based on the relative electronegativity of these groups, the four peaks can be assigned to –O– (epoxy group at 534.4 eV: marked as O1 peak), –OH (hydroxyl group at 533.1 eV: marked as O2 peak), –C=O (carbonyl group at 532.0 eV: marked as O3 peak), and –COOH (carboxyl group at 531.2 eV: marked as O4 peak) bonding features. O 1s core level spectra of rGO shows two distinct oxygen features (hydroxyl group marked as O2 and carbonyl group marked as O3). This interpretation is consistent with Lerf’s model^30^.

Through the Raman and HRPES results, we confirmed that they are consistent with previous results for GO and graphene, and thus we could confirm that the oxidation and reduction of rGO were successful. Next, we determined the oxidation (of 2-ATP) and reduction (of NB) activities of our rGO, GO, and graphene by using HRPES, their activities in the conversion of CO to CO_2_ by using residual gas analyzer, Orange II and methylene blue degradations by using UV-vis spectrophotometer, and their oxidation activities in aqueous solution by performing EC measurements.

### The Oxidation of 2-ATP and the Reduction of NB

**Figure 4:**
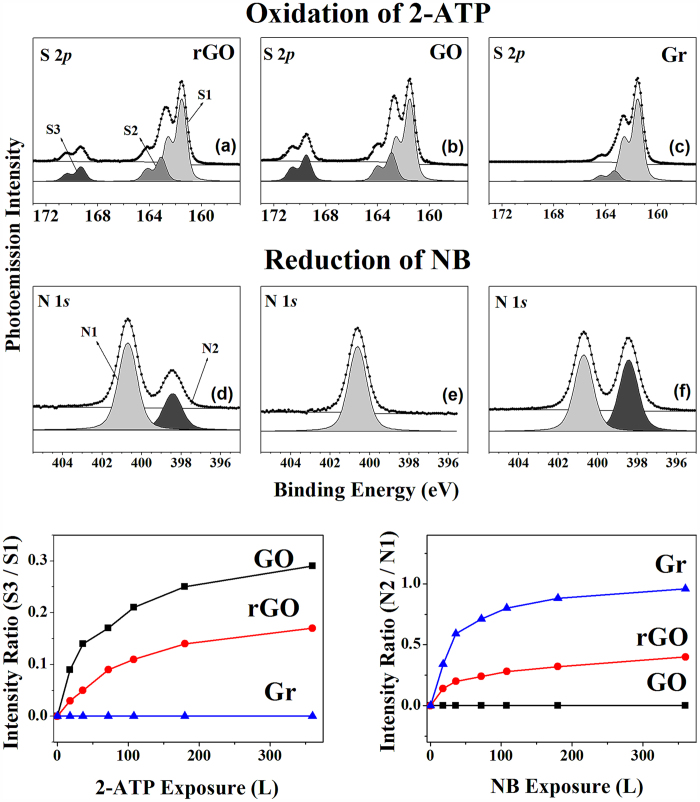
(Top panel) HRPES measurements of the S 2*p* core level peaks of (**a**) 360 L 2-ATP adsorbed on rGO, (**b**) 360 L 2-ATP adsorbed on GO, and (**c**) 360 L 2-ATP adsorbed on graphene at 300 K, and of the N 1*s* core level peaks of (**a**) 360 L NB adsorbed on rGO, (**b**) 360 L NB adsorbed on GO, and (**c**) 360 L NB adsorbed on graphene at 300 K. (Bottom panel) Plots of the ratios of S3 (sulfoxide group) to S1 (thiol group), which indicate the photocatalytic activities of the samples in the oxidation of 2-ATP (left panel) and those of the ratios of N2 (aniline group) to N1 (nitro group) via the reduction of NB (right panel) as a function of molecular exposure under 365 nm UV light.

We performed the photocatalytic oxidation of 2-ATP with the same amount of molecular oxygen and the reduction of NB with the same amount of molecular hydrogen adsorbed on the three distinct samples; both the oxidation and reduction reactions were carried out with exposure to 365 nm UV light. The oxidations of 2-ATP were monitored by determining the change in the intensity of the S 2*p* peak due to 2-ATP, which was obtained at a photon energy of 230 eV. Figure 4(a–c) show the surface-sensitive S 2*p* core level spectra after the co-exposure of 360 L 2-ATP to the same amount of oxygen on three distinct samples under 365 nm UV light illumination. These spectra contain three distinct 2*p*_3/2_ peaks at 161.5, 162.9, and 168.6 eV, each respectively corresponding to the C–SH unbounded state (denoted S1), the bounded state (denoted S2), and the sulfonic acid group (–SO_3_H) (denoted S3)^31^,^32^. It is known that sulfonic acid is formed from the oxidation of the thiol group in 2-ATP. We compared the oxidation activities of the three samples by monitoring the ratios of the intensities of peak S3 and S1. The increase in the level of sulfonic acid, i.e. the oxidation activities, are ordered as the following: graphene, rGO, and GO. During catalytic oxidation reaction using 2-ATP and molecular oxygen, we also confirm whether surface oxygen being included in GO or rGO is affected or not as we measured O 1s core level spectra. (see Figure S1) As shown in Figure S1, we can clearly confirm the oxygen function groups being included in GO or rGO do not change vividly and then they only can support the photocatalytic reaction of the exposed molecules. As a result, we can strongly insist that co-exposed 2-ATP and O_2_ react on GO or rGO under UV illumination.

We also performed the reduction of nitrobenzene (NB) in the presence of the three samples. Figure 4(d–f) show the surface-sensitive N 1*s* core-level spectra of NB on rGO, GO, and graphene on silicon substrates, obtained with HRPES at a photon energy of 460 eV. The N 1*s* core level spectra was obtained after introducing 360 L NB onto the rGO, GO, and graphene samples. As shown in Fig. 4(e), only a single nitrogen peak, the NB peak (marked N: -NO_2_), is evident, which indicates that no reduction occurs on GO as expected. In contrast, in the cases of rGO and graphene as shown in Fig. 4(d,f), there are two distinct nitrogen peaks, the NB peak (N1: -NO_2_) and the aniline peak (N2: -NH_2_). This indicates that the reduction reaction occurs on these samples. The ratios of the intensities of N2 to N1 were also plotted (see the bottom panel of Fig. 4). Our observations of the oxidation and reduction reactions on GO, rGO, and graphene with 2-ATP and NB confirm that GO can act as a good oxidative and reductive catalyst^33^,^34^.

As mentioned above, the bottom panels of Fig. 4 show the changes in the relative intensity as functions of 2-ATP (left panel) and NB (right panel) exposure under UV (365 nm) irradiation to enhance the photocatalytic activity. The variations in the ratios of the intensities of the S3 and S1 peaks (left panel) and those of the N2 and N1 peaks (right panel) reveal the photocatalytic oxidation and reduction activities of the samples. To be more specific, when the surface of GO is exposed to 360 L of 2-ATP, the S3/S1 ratio is 0.29. It is 1.70 times higher than the equivalent ratio for rGO (0.17). This higher S3/S1 ratio indicates that the oxidation of the adsorbed 2-ATP molecules is better facilitated on GO than on rGO or graphene. As the NB coverage increases from 0 L to 360 L, the intensity ratio (N2/N1) increases significantly from 0.00 to 0.96. The higher N2/N1 intensity ratio indicates that the reduction of the adsorbed NB molecules is better facilitated on graphene than on GO or rGO. Our observations of these reductions of NB on rGO, GO, and graphene confirm that graphene can act as a good reduction catalyst. In Fig. 4, we demonstrate the oxidation and reduction reactions in the presence of UV irradiation. To clear up the effect of UV irradiation, we also compared N 1s core level spectra of the three distinct carbon based materials in the presence and absence of UV illumination. (see Figure S2). As shown in Figure S2, we can clearly confirm that the reaction is not occurred or weak without UV illumination. In other word, during UV irradiation, reduction of nitrobenzene is being increased except GO. Hence, we can explain that there is the photocatalytic effect (oxidation or reduction).

### Conversion of CO to CO_2_

**Figure 5:**
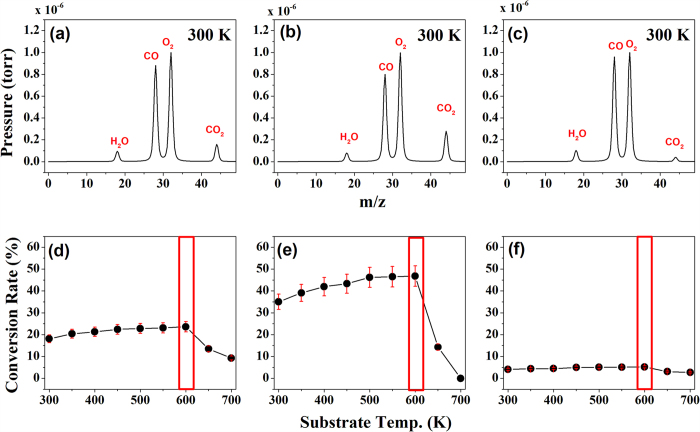
(Top panel) Mass spectra obtained from RGA of (**a**) rGO, (**b**) GO, and (**c**) graphene grown on silicon substrates after co-exposure to O_2_ and CO gas (1 × 10^−6^ torr) at 300 K. (Bottom panel) The variations with substrate temperature in the rates of conversion of CO to CO_2_ gas on (**d**) rGO, (**e**) GO, and (**f**) graphene.

A standard test for catalysts is the conversion of CO to CO_2_. Hence, we monitored the oxidation of CO to CO_2_ in the presence of the three distinct carbon materials by using RGA for various substrate temperatures in the range 300 K~700 K under UV irradiation. Figure 5(a~c) show the spectra obtained after CO and O_2_ exposures at 300 K to the three carbonaceous materials (rGO, GO, and graphene). Clearly, the rate of conversion of CO to CO_2_ on GO is larger than those on rGO and graphene, as expected because the various oxygen functional groups on GO facilitate CO oxidation. In addition, to determine the variations of substrate temperature in the CO oxidations of the three samples, we measured the conversion rates for various substrate temperatures (in 50 K increments from 300 K to 700 K) as shown in Fig. 5(d~f).

Two interesting points emerged from these measurements. First, the variation in the intensity of CO_2_ is dependent on that of CO even though the oxygen carriers on the carbon materials can assist CO oxidation; the low conversion rate on graphene confirms this phenomenon. Second, the conversion of CO to CO_2_ occurs stably in the range of 300 K to 600 K, as shown in Fig. 5(d~f). Above 600 K, this conversion decreases significantly and does not vary with temperature. In other words, CO oxidation does not occur on the surface of carbon materials when above 700 K, because substrate temperature of the three carbon based materials is enough high to decompose the molecules (CO and O_2_), the conversion process from CO and CO_2_ and the decomposed process can occur together! Hence, above 600 K, the conversion rate looks like decrement.

### Catalytic Activities: Orange II and methylene Blue degradation

**Figure 6:**
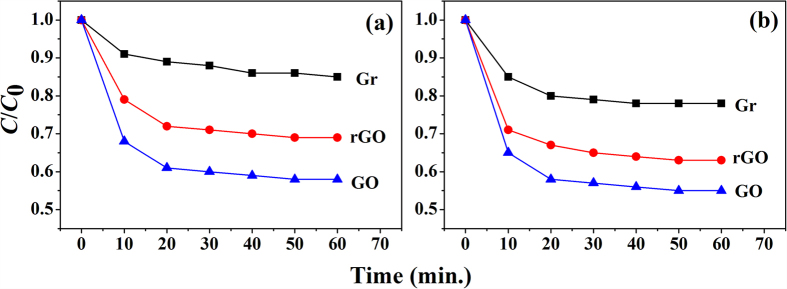
(**a**) Degradation of Orange II (0.15 mM) and (**b**) methylene Blue (0.15 mM) with GO, rGO, and graphene.

The catalytic oxidation of Orange II and methylene Blue was employed to probe the efficiency of the as prepared samples. As show in Fig. 6(a,b), the three carbon-based materials (rGO, GO, and graphene) present a negligible extent of dye removal. While slow degradation rate occurred, approximately 10~45% of the Orange II and methylene Blue was degraded in 60 minutes under the same condition. As shown in these plots, we found that the catalytic activity of GO is better than those of rGO and graphene as we expected; in detail, in case of GO, Orange II and MB can be degraded by nearly 45% in 60 minutes. Therefore, we could confirm that this result is well matched with our photocatalytic oxidation of 2-ATP and the conversion of CO to CO_2_ result. Consequentially, GO is better than others.

### Electrochemical redox reactions in the aqueous phase

**Figure 7:**
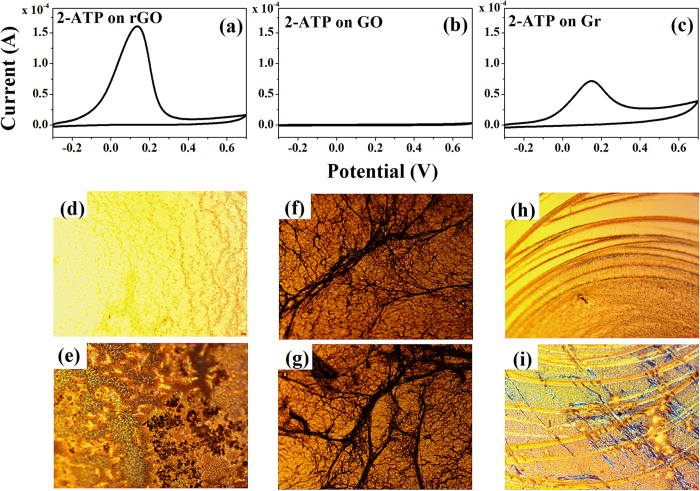
CVs of 20 mM 2-ATP were obtained on (**a**) rGO, (**b**) GO, and (**c**) graphene modified glassy carbon electrodes in 50 mM tris buffer at a scan rate of 100 mV/s. Optical microscopy images before and after the reaction: (**d,e**) rGO, (**f**,**g**) GO, and (**h,i**) graphene.

CVs were obtained to measure the electrocatalytic properties of rGO, GO and graphene with regards to the oxidation of 2-ATP. As shown in Fig. 7(b), there are no redox peaks for the GO modified electrode within the potential window, which indicates that GO has poor electrocatalytic activity for the electrochemical reaction of 2-ATP in an aqueous solution. In the solution phase, the buffer solution interacts with the functionalized oxygen on GO, so it is unlikely that the thiol group of 2-ATP will oxidize into sulfoxide, in contrast to the results under UHV conditions shown in Fig. 4(b).

Further, there is a high oxidation current at 0.15 V in the CV results for rGO (see Fig. 7(a)) and graphene (see Fig. 7(c)). These peaks for rGO and graphene have a bell-like shape, which is typical of the CVs of adsorbed species. From these results, we conclude that 2-ATP is adsorbed on rGO and graphene-modified electrode surfaces, and that this adsorbed 2-ATP can be oxidized because of the electrocatalytic activities of rGO and graphene. In addition, there are no reduction peaks, which means that the electrochemical oxidation of 2-ATP is irreversible. In contrast to the rGO and graphene samples, there are no oxidation peaks in the case of the GO sample. The electrochemical oxidation of 2-ATP is prevented by the oxygen species in GO. In addition, as shown in Fig. 7(d~i), we can clearly observe the changes in the electrode conditions before and after reaction for the three carbonaceous samples. There are no electrocatalytic changes for GO although we conducted the CV measurements before (see Fig. 7(f)) and after 2-ATP activation (see Fig. 7(g)), which indicates that it does not show any oxidation reaction in aqueous solutions as we confirm the change of electrode before and after the reaction. On the contrary, we can clearly observe the scratch of electrode in the cases of rGO (see Fig. 7(d,e)) and graphene (see Fig. 7(h,i)). Thus we again can confirm the comparative results during three distinct carbon based materials (rGO, GO, and graphene) despite the trend in the EC results being the opposite to that found under UHV conditions because of the blocking of aqueous solution.

## Conclusion

In conclusion, we have successively fabricated GO (oxidation) and graphene (reduction) from rGO by controlling the reaction times. In order to compare the catalytic activities (in oxidation and reduction processes) of the three carbonaceous materials, we conducted oxidation of 2-aminothiophenol and reduction of nitrobenzene in their presence and monitored the reactions by using high-resolution photoemission spectroscopy (HRPES). In addition, we also determined the rates of conversion of CO to CO_2_ in the presence of these catalysts by performing residual gas analyzer (RGA), Orange II and methylene blue degradation and electrochemistry (EC) measurements in aqueous solution, as well as by obtaining cyclic voltammograms and determining the change in the condition of the electrodes before and after the oxidation of 2-ATP. We found that we can successively fabricate GO (oxidation) and graphene (reduction) from rGO by controlling the oxidation or reduction process time. Furthermore, we characterized the morphologies, electronic properties, and electrochemistry of the catalysts by using SEM, TEM, Raman spectroscopy, HRPES, RGA, UV-Vis spectrophotometry, and EC measurements. We confirmed that GO can act as a catalyst for oxidation reactions and graphene can act as a catalyst for reduction reactions.

## Experimental Methods

### Preparation of GO and graphene

GO was obtained from rGO by using the Hummers’ method^25^,^35^,^36^. Based on the Hummers’ method, we made slight modifications by exposing the rGO nanoparticles to 3 M KMnO_4_, H_2_SO_4_, and 0.5 M NaNO_3_ as we controlled the reaction time. Graphene fabricated from rGO was carried out by adding hydrazine (1.0 mL) into the dispersion of rGO (50 mg of rGO in 100 mL of water)^37^. After having sonicated for 1 h and kept stirring for 24 h at 60 °C for the fabricated GO and graphene, we characterized with Raman and C 1*s* core-level spectroscopy (see Fig. 3) after doing a spin-coated process onto silicon substrates.

### Materials

2-aminothiophenol (2-ATP; Sigma Aldrich, purity, 99.5%) and nitrobenzene (NB; Sigma Aldrich, purity, 99.0%), Orange II (Sigma Aldrich, purity, 99.5%), methylene blue (Sigma Aldrich, purity, 99.5%), carbon monoxide (CO, 99.95%) and molecular oxygen (O_2_, 99.99%) gas were purified by turbo-pumping to remove impurities prior to dosing onto the GO, rGO, and graphene samples grown on silicon substrates. A direct dozer, controlled by using a variable leak valve, was used to dose the substrates.

### Characterizations

Scanning electron microscopy (SEM) images of the samples were obtained by using a field-emission scanning electron microscope (JEOL JSM-7600F) operated at an acceleration voltage of 15 kV and a field-emission transmission electron microscope (JEOL JEM-2100F(HR)) operated at an accelerating voltage of 200 kV. Raman spectra of the samples was collected by using a home-built system equipped with an Ar^+^ ion laser (Spectra-Physics Stabilite 2017) as an excitation source, a spectrometer (Horiba Jobin Yvon TRIAX 550), and a CCD detector (Horiba Jobin Yvon Symphony) cooled to −133 °C. The wavelength of the incident excitation beam was 514.5 nm. HRPES experiments were performed at the 8A2 beamline at Pohang Accelerator Laboratory (PAL) which is equipped with an electron analyzer (SES100, Gamma Data Scienta). The S 2*p*, C 1*s*, and N 1*s* core level spectra were obtained by using photon energy of 230, 340, and 460 eV respectively. The binding energy of the core level spectra was determined with respect to the binding energy of the clean Au 4*f* core level for the same photon energy. All spectra were recorded in the normal emission mode. The photoemission spectra were carefully analyzed by using a standard nonlinear least squares fitting procedure with Voigt functions^38^. Mass spectrometric analysis by RGA was carried out under ultra-high vacuum conditions with a Hiden RC 301 (mass range ~300 amu) system that can be operated in positive-ion mode to detect CO, O_2_, and CO_2_ gas intensities. The experiments for degradation of orange II and methylene blue (MB) in water by advanced oxidation technology were conducted in a 500 mL beaker under 25 °C thermostat water baths with constant magnetic stirring. Typically, with Orange II (0.15 mM) and MB (0.15 mM), Intensity variation of GO, rGO, and graphene was obtained at regular intervals, joined immediately with 1 mL methanol as the inhibitor, and then filtered. The real-time concentration was measured in an UV−vis spectrophotometer (UV−2600, SHIMADZU) at 484 nm. The electrochemical experiments were performed by using a CHI617B potentiostat (CH Instruments, Austin, TX) with a three-electrode cell placed in a Faraday cage. A 2 mm diameter glassy carbon electrode was used as the working electrode and a 1 mm diameter Pt wire was used as the counter electrode. The reference electrode was Ag/AgCl (3 M KCl). All working electrodes were polished with alumina (0.05 μm) paste on micro-cloth pads (Buehler, Lake Bluff, IL) prior to use. The drop casting method was performed on glassy carbon electrodes to measure the electrocatalytic properties of GO, rGO, and graphene. The samples (5 μL) were placed on glassy carbon electrodes, and then were stored in an oven at 40 °C for 6 h. All cyclic voltammograms (CVs) were obtained in 50 mM tris buffer (pH 9.0) at a scan rate of 100 mV/s^39^,^40^.

## Additional Information

**How to cite this article**: Lee, H. *et al*. Comparative Study of the Catalytic Activities of Three Distinct Carbonaceous Materials through Photocatalytic Oxidation, CO Conversion, Dye Degradation, and Electrochemical Measurements. *Sci. Rep.* **6**, 35500; doi: 10.1038/srep35500 (2016).

## Supplementary Material

Supplementary Information
