# Supplementary material for: Comparative Study of the Catalytic Activities of Three Distinct Carbonaceous Materials through Photocatalytic Oxidation, CO Conversion, Dye Degradation, and Electrochemical Measurements
Source: Sci Rep. 2016 Oct 20;6:35500. doi: 10.1038/srep35500 (PMC5071860; doi:10.1038/srep35500)
Supplement: Supplementary Information [file srep35500-s1.pdf]

Supporting Information

Comparative Study of the Catalytic Activities of Three Distinct Carbonaceous Materials through Photocatalytic Oxidation, CO Conversion, Dye Degradation, and Electrochemical Measurements

Hangil Lee[*,1] *Yeonwoo Kim,*[2] *Min Ji Kim,*[1] *Ki-jeong Kim,*[3] *Byung-Kwon Kim*[1]

[1] Department of Chemistry, Sookmyung Women's University, Seoul 140-742, Republic of Korea

[2] Molecular-Level Interfaces Research Center, Department of Chemistry, KAIST, Daejeon 305-701, Republic of Korea

[3] Beamline Research Division, Pohang Accelerator Laboratory (PAL), Pohang 790-784, Republic of Korea

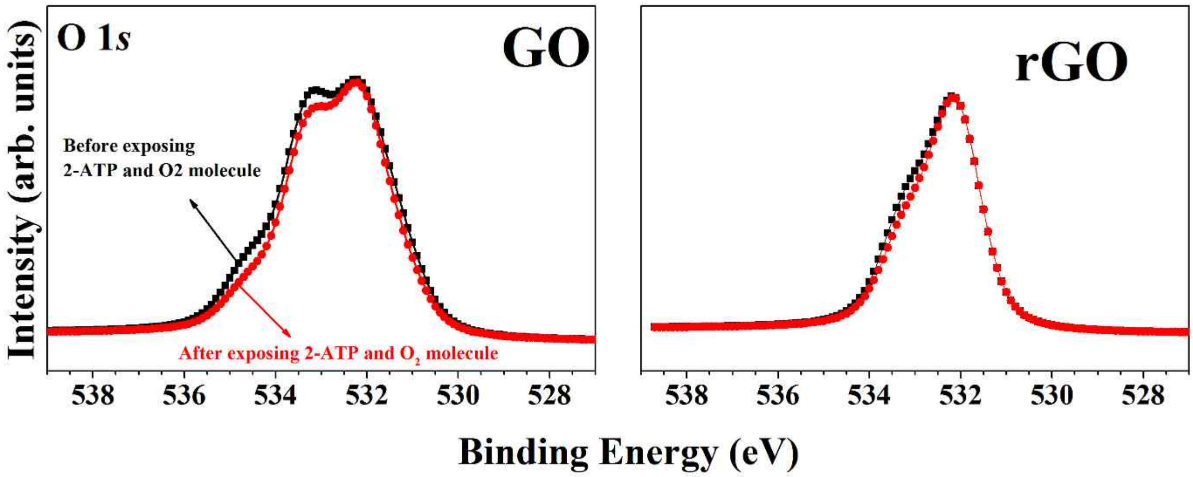

Figure S1. (Left panel) O 1*s* core level peaks of GO before exposing molecule (black color) and after exposing molecules (red color) obtained at 300 K in the presence of UV irradiation. (Right panel) O 1*s* core level peaks of rGO before exposing molecule (black color) and after exposing molecules (red color) obtained at 300 K in the presence of UV irradiation.

Figure S1 displays O 1*s* core level spectra of the three distinct carbon based materials during catalytic oxidation reaction using 2-ATP and molecular oxygen to confirm whether surface oxygen being included in GO or rGO is affected or not. As shown in Figure S1, we can clearly confirm the oxygen function groups being included in GO or rGO do not change vividly. As a result, we can strongly insist that co-exposed 2-ATP and O_2 react on GO or rGO under UV illumination and then they only can support the photocatalytic reaction of the exposed molecules.

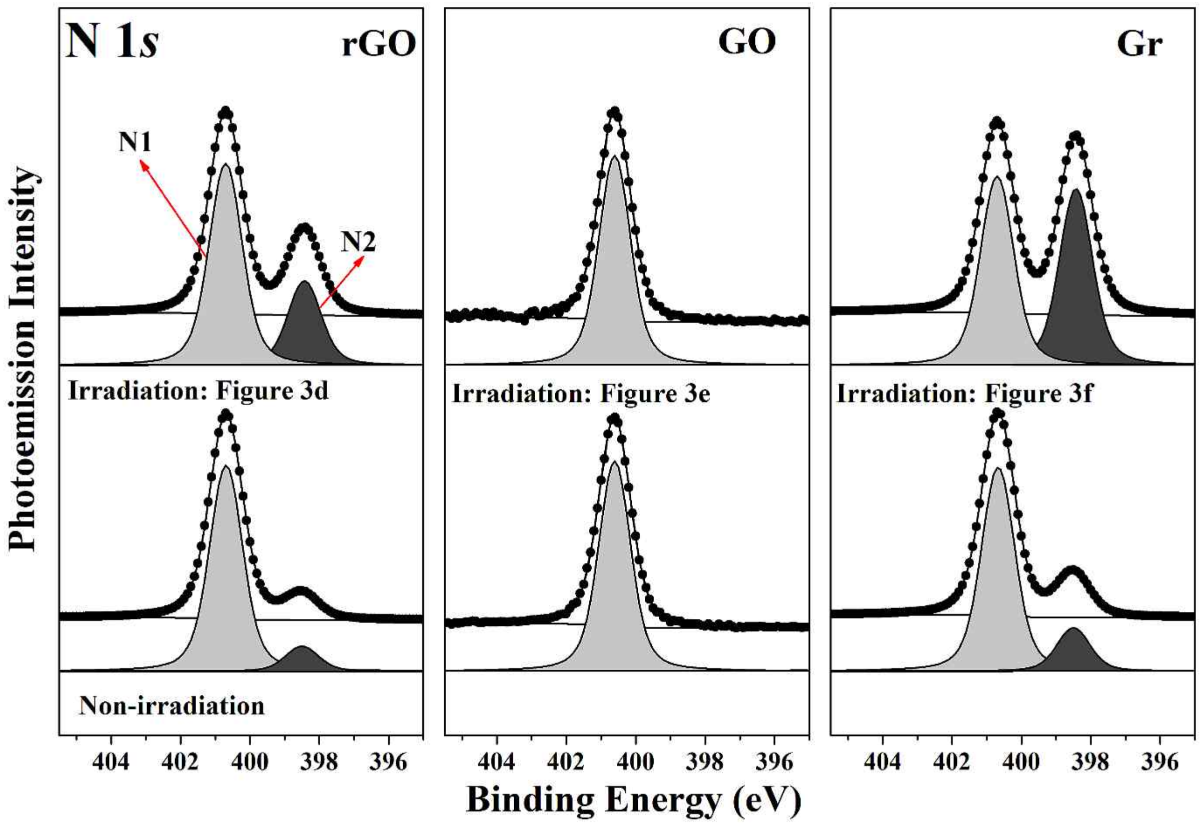

Figure S2. (Top panel) HRPES measurements of the N 1*s* core level peaks of (a) 360 L 2-ATP adsorbed on rGO, (b) 360 L 2-ATP adsorbed on GO, and (c) 360 L 2-ATP adsorbed on graphene at 300 K in the presence of UV irradiation. (Bottom panel) the N 1*s* core level peaks of (a) 360 L NB adsorbed on rGO, (b) 360 L NB adsorbed on GO, and (c) 360 L NB adsorbed on graphene at 300 K in the absence of UV irradiation.

Figure S2 shows N 1*s* core level spectra of the three distinct carbon based materials in the presence and absence of UV irradiation to confirm the effect of UV illumination. As shown in figures, we can clearly confirm that during UV irradiation, reduction of NB is being increased except GO.
